# Drug-induced Parkinsonism: A strong predictor of idiopathic Parkinson’s disease

**DOI:** 10.1371/journal.pone.0247354

**Published:** 2021-03-01

**Authors:** Sohyun Jeong, Hyemin Cho, Yun Joong Kim, Hyeo-Il Ma, Sunmee Jang

**Affiliations:** 1 Marcus Institute for Aging Research at Hebrew SeniorLife, Boston, Massachusetts, United States of America; 2 Department of Medicine, Beth Israel Deaconess Medical Center and Harvard Medical School, Boston, Massachusetts, United States of America; 3 College of Pharmacy and Gachon Institute of Pharmaceutical Sciences, Gachon University, Incheon, Korea; 4 Department of Neurology, Yonsei University College of Medicine, Yongin Severance Hospital, Yongin-si, Gyeonggi-do, Korea; 5 Department of Neurology, Hallym University College of Medicine, Anyang, Gyeonggi-do, Korea; Istituto Di Ricerche Farmacologiche Mario Negri, ITALY

## Abstract

**Background:**

Although Idiopathic Parkinson’s disease (IPD) develops in considerable patients with drug-induced Parkinsonism (DIP), the association hasn’t been well defined. We aimed to evaluate the underlying association and risk factors of DIP and IPD.

**Methods:**

A retrospective cohort study using National Health Insurance Claims data in 2011–2016 was conducted. New-onset DIP patients in 2012 were selected and matched with active controls having diabetes mellitus at a 1:4 ratio by age, sex, and Charlson’s Comorbidity Index score. Comorbidity, causative drugs, and prescription days were evaluated as covariates.

**Results:**

A total of 441 DIP were selected. During the 4-year follow up, 14 IPD events in the DM group but 62 events in the DIP group were observed (adjusted hazard ratio, HR: 18.88, 95% CI, 9.09–39.22, adjusting for comorbidities and causative drugs). IPD diagnosis in DIP was observed high in males compared to females (15.58/13.24%). The event was the most within the 1^st^ year follow-up, mean days 453 (SD 413.36). Subgroup analysis in DIP showed calcium channel blocker (verapamil, diltiazem, and flunarizine) was significantly associated with increased IPD risk (HR: 2.24, 95% CI, 1.27–3.93).

**Conclusion:**

Increased IPD in DIP patients might not be from the causal toxicity of antidopaminergic effects but from a trigger by the causative drugs on the DIP patients who already had subclinical IPD pathology. DIP can serve as a strong proxy for IPD incidence. Subjects who develop DIP should be monitored carefully for potential IPD incidence.

## Introduction

Drug-induced Parkinsonism (DIP) is the most serious iatrogenic movement disorder in the elderly as it increases the risk of gait dysfunction, falls, assisted living condition and considerably decreases the daily functioning and quality of life [1]. A recent 2016 report from the Rochester Epidemiology Project reported that the incidence of both the Idiopathic Parkinson’s disease (IPD) and Parkinsonism were increased significantly over the 30 years. The relative risk (RR) for IPD was 1.35 per decade (95% CI, 1.10–1.65), and the RR for Parkinsonism was 1.24 per decade (95% CI, 1.07–1.44) [2]. The average annual incidence rate of DIP over 30 years in Olmsted County, Minnesota was 3.3 per 100,000 person-years overall (108 patients), 2.1 in men (33 patients), and 4.3 in women (75 patients). DIP was the most common type of Parkinsonism, accounting for 11 of 15 cases (73.3%) in the youngest age group (0–39 years) [1].

The risk factors for DIP include aging, female sex, anti-dopaminergic potency and length of causative drug use, pre-existing extrapyramidal signs, and familial or genetic predisposition [3]. The main etiology of DIP is from diminished D2 receptor stimulation, which occurs primarily through D2 receptor blockade by antipsychotics and related drugs [4].

In general, distinct differences between DIP and IPD exist by molecular imaging techniques; the estimated number of presynaptic dopamine-secreting neurons is diminished in IPD, but normal in DIP [4]. In general, the symptoms of Parkinsonism resolve in 6 months in DIP [5], and 70% of patients usually recover within a few months after withdrawal of the causative drugs. However, Parkinsonism persists in the remaining DIP patients [6, 7], and a considerable proportion of DIP patients is diagnosed with IPD [8, 9]. This phenomenon has evoked concerns about whether neuroleptic harmful exposure of anti-dopaminergic drugs (presented as DIP) would cause IPD.

Kim et al presented 2 DIP patients who initially presented normal dopamine transporter (DAT) activity and reached full remission, but experienced recurrence with decreased DAT activity at a 2-year follow-up scan [10]. Burn & Brook also reported 5 patients who had normal DAT imaging at DIP diagnosis did not experience full remission until 2 years [11]. Foubert-Samier et al [12] conducted a prospective study and demonstrated a long-term neuroleptic exposure might be a risk factor for IPD as well. Based on these findings, one theory supports some dopamine receptor blockers have direct toxic effects on neurons by inhibiting mitochondrial respiratory function and contribute to the irreversible cell death [13] or pathway deficits in nigrostriatal dopamine area [14].

However, another theory argues that a significant loss of dopaminergic neurons already exists in DIP patients, and the causative drugs accelerate the development of IPD [7, 15]. Adding on this theory, a recent study showed that 6 out of 7 patients with DIP had pathological findings compatible with an underlying IPD [16], which implicates dopamine blocking agents can unmask preclinical PD.

In the context of these conflicting findings, we aimed to evaluate the association and potential risk factors of DIP diagnosis and IPD incidence using a nationwide population-based health claims database.

## Materials and methods

### Study design and database

A retrospective cohort design was adopted. The cohort comprised all DIP patients in the National Health Insurance Claims Database combined with pharmacy prescription claims data in 2010–2016; the year 2010 and 2011 were used for the screening, 2012 for enrollment, and 2013–2016 for the 4-year follow-up. The national health insurance program in Korea provides universal coverage for the entire population, including roughly 50 million Koreans, so the diagnosis and therapeutic trends and characteristics of IPD and DIP could be evaluated systematically.

This study was approved by Hallym University Sacred Heart Hospital Institutional Review Board (IRB No. 2016–1081). We acquired the data through a form that deleted individual identities and only received the items pertaining to this research purpose, IRB did not require us to obtain informed consent from individual patients. All procedures performed in this study were conducted in accordance with the ethical standards of the institutional and/or national research committee and with the 1964 Helsinki declaration and its later amendments or comparable ethical standards.

### Study design and subject definition

DIP subjects were defined as patients who 1) were 50–100 years of age, and 2) had a diagnosis code of DIP (ICD-10: G21.1), 3) had prescriptions of causative drugs within 1 year before DIP diagnosis. DIP is regarded as a sort of drug adverse events, and the diagnosis code is used when the physicians made sure Parkinsonism related symptoms are disappeared or reduced after the probable drugs are interrupted, which makes the code having high ascertainment.

As a comparison, we adopted an active comparator (patients with Diabetes Mellitus, DM) to avoid confounding bias common in pharmacoepidemiologic studies such as the healthy initiator bias in the selection of controls where control subjects might be too healthy compared to the normal population [17–19]. Since accumulating prospective cohort studies showed a possible association between DM and IPD incidence that pre-existing DM might be a risk factor for the development of IPD [20], we chose DM as an active control. DM subjects were defined as new-onset patients who had ICD-10 codes (E11, E12, E13, E14) along with anti-diabetes prescription. DM patients were matched to the exposed group (DIP) in 4:1 ratio by a propensity score derived from sex, age, and Charlson Comorbidity Index (CCI).

To include new-onset subjects, we excluded patients who had a diagnosis code for DIP or IPD in 2010 and 2011. The index dates (cohort entry date) for the DIP and DM group were the date of the first DIP or DM diagnosis code that appeared in claim data, respectively.

IPD patients were defined as those who had v-124 code, which is used for specific beneficiary insurance claims for IPD treatment in Korea. Since the v-124 code yields a major benefit to IPD patients who only need to pay 10% of all medical expenditures, assigning this code to a patient requires careful and complicated documentation. To assign the v-124 code to the patients, diagnostic MRI findings and major Parkinsonism syndromes including tremor, bradykinesia, rigidity, and postural instability should be examined and confirmed by the neurologists. Therefore, we were able to use the v-124 code for IPD ascertainment.

The event dates for both the DIP and the DM were defined as the first diagnosed date of IPD (v-124). We evaluated all the included patients until the last date of four-year follow-up or follow-up loss or death dates whichever comes first. To assess the comparison between pure DIP and DM patients, when DIP incidence happened in the DM group, they were followed up until DIP incidence happened (censoring was applied).

### Definition of confounders

Age, sex, CCI scores, and comorbidities in the previous year and DIP-inducing (causative) drugs taken within 12 months before DIP diagnosis were assessed.

Drugs known to induce Parkinsonism included typical and atypical antipsychotics, dopamine depleters, dopamine synthesis inhibitors, calcium channel blockers (CCB), antiepileptics, antiemetics, a mood stabilizer (lithium), antiarrhythmics, antidepressants, and an immunosuppressant (cyclosporin), as proven by previous research [9]. They were reclassified into four categories by their potency for D2 receptor blockade (S1 Table).

Diseases that could affect IPD incidence but were not included in the CCI score calculation were assessed as variables (comorbidities). They were dementia [21, 22], depression [23], other neurodegenerative diseases [24], and hypertension [25]. The ICD-10 codes defining these diseases are provided in S2 Table.

### Statistical analysis

Baseline characteristics were compared by the t-test for quantitative and the chi-square test for qualitative variables. Cox proportional hazard regression analysis was performed to evaluate the risk of IPD incidence in both groups. The hazard ratios (HRs) and the corresponding 95% CIs were estimated, adjusting for possible confounders. A sub-group analysis in the DIP group to identify potential risk factors associated with IPD was conducted using Cox proportional hazard regression analysis. All analyses were performed using SAS version 9.4 (SAS Institute, Cary, NC, USA).

## Results

### Study subjects

**Fig 1:**
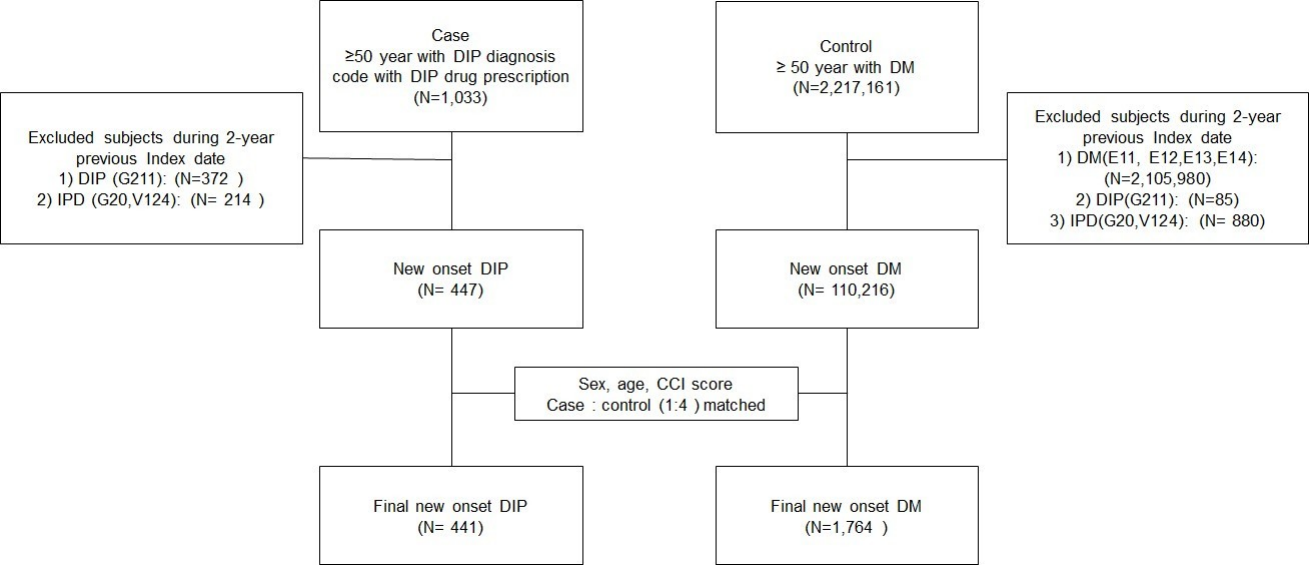
Flow diagram of subject selection and matching.

A total of 441 patients satisfied the inclusion criteria of DIP, and 1,764 DM patients were randomly matched by a propensity score calculated by age, sex, and CCI score at 1:4 ratio (Fig 1).

At baseline, female patients constituted 64.49% and the 70-79-years group constituted 43.67%, which was the highest proportion of the total subject. CCI score ranging 1–2 was 50.43% having the highest number of subjects. Hypertension (43.13%) was the highest comorbidity followed by depression (28.44%) and dementia (11.97%) in the total subject, which showed the same trend in both groups.

The causative drugs taken within 12 months before the DIP diagnosis were identified in 34.01% in the DM subjects. The most commonly prescribed causative drugs were class III (anti-emetics) in both groups (82.09%: DIP group, 29.76%: DM group). The next was class I (antipsychotics and others) for the DIP group (36.5%) and class IV (antidepressants and others), for the DM group (8.73%). Mean prescription days were 60.81(122.45) days for DM vs. 261.2 (190.46) days for the DIP group *(p <* .*0001)*. In the DM group, the subject number who had prescription day ≥ 70% of the previous 1 year was 9.41% whereas it was 46.03% in the DIP group (Table 1).

**Table 1:** Baseline characteristics of DM and DIP subjects.

Category		Subjects						*P value*
DM, N (%)		DIP, N (%)		Total, N (%)				
Sex	Male	629	35.66	154	34.92	783	35.51	0.772
	Female	1135	64.34	287	65.08	1422	64.49	
Age	50–59 y	275	15.59	70	15.87	345	15.65	0.995
	60–69 y	421	23.87	104	23.58	525	23.81	
	70–79 y	769	43.59	194	43.99	963	43.67	
	≥80 y	299	16.95	73	16.55	372	16.87	
	Mean age (SD)	70.46 (9.39)		70.44 (9.43)		70.46 (9.40)		0.959
CCI score	0	607	34.41	154	34.92	761	34.51	0.877
	1–2	894	50.68	218	49.43	1112	50.43	
	≥3	263	14.91	69	15.65	332	15.06	
	Mean CCI (SD)	1.19 (1.31)		1.18 (1.21)		1.19 (1.29)		0.863
Co-Morbidity	Dementia	214	12.13	50	11.34	264	11.97	0.646
	Depression	502	28.46	125	28.34	627	28.44	0.962
	Hypertension	771	43.71	180	40.82	951	43.13	0.273
	Neuro-degenerative disease	46	2.61	14	3.17	60	2.72	0.513
DIP-inducing drugs** (causative drugs)	Class I (antipsychotics)	101	5.73	161	36.51	262	11.88	< .0001
	Class II (calcium channel blockers)	107	6.07	65	14.74	172	7.80	< .0001
	Class III (antiemetics)	525	29.76	362	82.09	887	40.23	< .0001
	Class IV (antidepressants)	154	8.73	110	24.94	264	11.97	< .0001
	Total	600	34.01	441	100.00	1041	47.21	< .0001
	None	1164	65.99	0	0.00	1164	52.79	-
Total prescription days in 1 year previous Index date	0%	1164	65.99	0	0.00	1164	52.79	< .0001
	0–30%	263	14.91	123	27.89	386	17.51	
	30–70%	171	9.69	115	26.08	286	12.97	
	> = 70%	166	9.41	203	46.03	369	16.73	
	Mean days	60.81(122.45)		261.2 (190.46)		100.89 (160.19)		< .0001
Total		1764	100.00	441	100.00	2205	100.00	

**DIP inducing drugs were multiple counted if subjects had different prescriptions during screening period.

### Overall IPD risk between DIP and DM

DIP group had much higher IPD events than the DM group; 62/441(14.06%) in DIP vs. 14/1764 (0.79%) in DM. (*p <* .*0001*) The Cox proportional hazard regression analysis found a 17.90 times greater hazard of IPD in the DIP group than in the DM group (crude HR: 17.90, 95% CI, 10.02–31.97). When comorbidities and causative drugs were adjusted, the HR increased to 18.88 (adjusted HR: 18.88; 95 CI, 9.09–39.22).

Sex, age, and comorbidity did not significantly affect IPD incidence in both groups. Different levels of CCI scores did not significantly affect IPD incidence in either group. When the higher was the score, the more incidence of IPD was detected in both groups.

The neurodegenerative disease presented the highest IPD incidence (21.43%) but the other comorbidities showed similar incidences around 15–16% in the DIP group. In the DM group, dementia presented the highest IPD incidence yielding 2.34% similar to neurodegenerative disease (2.17%).

Among the causative drug classes, class II (CCB) was significantly associated with IPD incidence in the DIP group (*p <* .*0001*). In the DIP groups, IPD incidence was the highest in Class II (27.69%), but Class III (13.81%) and Class IV (13.64%) showed similar incidences. On the contrary, Class I (1.98%) and Class IV (1.95%) had a similarly high incidence of IPD in the DM group. The mean duration of taking causative drugs before DIP diagnosis in each group was not significantly different (Table 2).

**Table 2:** IPD incidence between DM and DIP subjects.

Category		DM (N = 1,764)						DIP (N = 441)					
		Non-IPD		IPD		IPD N/row total N	*p-value*	Non-IPD		IPD		IPD N/row total N	*p-value*
		N	%	N	%	%		N	%	N	%	%	
Total, N		1750	99.21	14	0.79	0.79		379	85.94	62	14.06	14.06	
Mean follow-up days		1,211 (484.90)		832 (463.23)		1,296(485)	0.23	1,296(380.62)		453 (413.53)		1,178 (484)	< .0001
Sex	Male	624	35.37	5	0.28	0.79	1.00	130	29.48	24	5.44	15.58	0.50
	Female	1126	63.83	9	0.51	0.79		249	56.46	38	8.62	13.24	
Age	50–59 y	275	15.59	0	0.00	0.00	0.30	64	14.51	6	1.36	8.57	0.26
	60–69 y	418	23.70	3	0.17	0.71		85	19.27	19	4.31	18.27	
	70–79 y	760	43.08	9	0.51	1.17		165	37.41	29	6.58	14.95	
	≥80 y	297	16.84	2	0.11	0.67		65	14.74	8	1.81	10.96	
	Mean (SD)	70.43 (9.41)	73.93 (7.05)	70.46 (9.40)	0.17	70.36 (9.62)	70.87 (8.24)	70.44 (9.43)	0.70
CCI score, mean	0	605	34.30	2	0.11	0.33	0.17	135	30.61	19	4.31	12.34	0.43
	1–2	886	50.23	8	0.45	0.89		188	42.63	30	6.80	13.76	
	≥3	258	14.63	4	0.23	1.53		56	12.70	13	2.95	18.84	
	Mean (SD)	1.19 (1.31)		1.64 (1.08)		1.19 (1.31)	0.19	1.17 (1.21)		1.26 (1.21)		1.18 (1.21)	0.58
*Co-Morbidity	Dementia	209	11.85	5	0.28	2.34	0.01	42	9.52	8	1.81	16.00	0.68
	Depression	496	28.12	6	0.34	1.20	0.23	105	23.81	20	4.54	16.00	0.46
	Hypertension	763	43.25	8	0.45	1.04	0.31	152	34.47	28	6.35	15.56	0.45
	Neuro-degenerative	45	2.55	1	0.06	2.17	0.29	11	2.49	3	0.68	21.43	0.42
**Causative drugs taken 1 year before DIP diagnosis	Class I	99	5.61	2	0.11	1.98	0.17	144	32.65	17	3.85	10.56	0.11
	Class II	107	6.07	0	0.00	0.00	0.34	47	10.66	18	4.08	27.69	0.00
	Class III	518	29.37	7	0.40	1.33	0.10	312	70.75	50	11.34	13.81	0.75
	Class IV	151	8.56	3	0.17	1.95	0.09	95	21.54	15	3.40	13.64	0.88
	Total defined	592	33.56	8	0.45	1.33	0.07	379	85.94	62	14.06	14.06	-
	None	1158	65.65	6	0.34	0.34		0	0	0	0	0.00	
#Total prescription days in 1 year prior to the Index date	0%	1158	65.65	6	0.34	0.52	0.27	0	0.00	0	0.00	-	0.11
	<30%	260	14.74	3	0.17	1.14		101	22.90	22	4.99	17.89	
	30%-70%	168	9.52	3	0.17	1.75		96	21.77	19	4.31	16.52	
	> = 70%	164	9.30	2	0.11	1.20		182	41.27	21	4.76	10.34	
	Mean days	60.41 (122.24)		110.5 (141.71)		60.8 (122.5)	0.13	268.67 (193.08)		215.55 (167.84)		268.2 (190.5)	0.18

*Co-morbidities were multiple counted if subjects had different ICD-10 codes during screening period.

** Dichotomous evaluation of having prescription (yes or no) were done and multiple counted if subjects had different prescriptions during screening period.

#Total sum of durations of all available prescriptions (duplicate prescriptions were allowed and overlapping period was not subtracted).

The total follow-up days for DIP and DM was 1,178.08 (SD 484.12) and 1,208.66 (SD 485.78) (*p = 0*.*237*), respectively. During the 4-year follow-up, 446 subjects died in the DM group but 77 subjects died in the DIP group (*p = 0*.*0005*) (S3 Table).

### IPD risk by the year in age and sex groups

**Fig 2:**
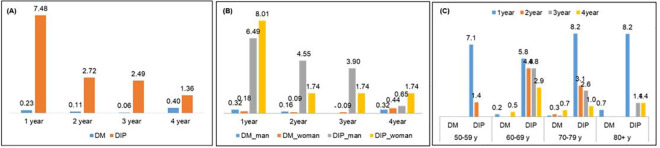
Age and sex stratified IPD incidence by the year.

Aging is the strongest risk factor for IPD [26] and sex difference in IPD has been well characterized [27], so we evaluated IPD incidence by the year in age and sex groups. When IPD events by each year were compared in both groups, IPD incidence was the highest in the 1^st^ year with a dramatic reduction thereafter in the DIP group. However, the DM group did not show a trend and it was the highest at the 4^th^ year, suggesting DIP events triggered IPD diagnosis in DIP group whereas IPD diagnosis in DM group was increased with the time (aging) (Table 3, Fig 2A).

**Table 3:** Age and sex stratified IPD incidence by the year.

Category		IPD, N (%)											
		DM						DIP					
		Total DM	1year	2year	3year	4year	Total IPD	Total DIP	1year	2year	3year	4year	Total IPD
Sex	Male	629 (100)	2(0.32)	1(0.16)	0	2(0.32)	5(0.79)	154(100)	10(6.49)	7(4.55)	6(3.90)	1(0.65)	24(15.58)
	Female	1135(100)	2(0.18)	1(0.09)	1(0.09)	5(0.44)	9(0.79)	287(100)	23(8.01)	5(1.74)	5(1.74)	5(1.74)	38(13.24)
Age	50–59 y	275 (100)	0	0	0	0	0	70 (100)	5 (7.14)	1(1.43)	0	0	6(8.57)
	60–69 y	421 (100)	1(0.24)	0	0	2(0.48)	3(0.71)	104(100)	6(5.77)	5(4.81)	5(4.81)	3(2.88)	19(18.27)
	70–79 y	769 (100)	1(0.13)	2(0.26)	1(0.13)	5(0.65)	9(1.17)	194(100)	16(8.25)	6(3.09)	5(2.58)	2(1.03)	29(14.95)
	≥80 y	299 (100)	2(0.67)	0	0	0	2(0.67)	73(100)	6(8.22)	0	1(1.37)	1(1.37)	8(10.96)
	Total	1764 (100)	4(0.23)	2(0.11)	1(0.06)	7(0.40)	14(0.79)	441(100)	33(7.48)	12(2.72)	11(2.49)	6(1.36)	62(14.06)

Abbreviation; IPD, Idiopathic Parkinson’s Disease; DM, Diabetes Mellitus; DIP, Drug Induced Parkinsonism.

Both sexes presented the same proportion of IPD incidence in the DM group (0.79%). Although it was not significant, males had higher proportion of IPD incidents than females in the DIP group (15.58% vs. 13.24%) (Table 3, Fig 2B).

IPD occurred highest in the 60-69-years age category in DIP (18.27%) but 70-79- years age group in DM (1.17%) in the follow-up period. In both groups, the 50-59-years age group developed the fewest IPD events; the DIP group (8.57%) and the DM group (0%). The highest IPD events in all the age groups occurred in the first year of follow-up in the DIP group (Table 3, Fig 2C).

### Subgroup analysis: Risk factors associated with IPD diagnosis in DIP

The risk factors associated with IPD development were evaluated by a Cox proportional hazard regression model, considering the death events in the DIP group. Sex, age group, and comorbid condition were not significantly associated with IPD. Only Class II (CCB) presented a significantly increased risk of IPD (HR: 2.24, 95% CI: 1.28–3.93) (Table 4).

**Table 4:** Subgroup analysis: Risk factors of IPD incidence in DIP subjects.

Variables	Reference	Hazard Ratio	95% CI	
Male	Female	1.32	0.782	2.228
65–74 year	<65 year	1.129	0.564	2.257
≥ 75	<65 year	1.024	0.487	2.152
CCI continuous		1.174	0.659	2.092
Dementia	No	1.298	0.572	2.947
Depression	No	1.201	0.686	2.104
Hypertension	No	1.307	0.777	2.199
Neurodegenerative disease	No	1.516	0.466	4.937
Class I	No	0.554	0.271	1.13
Class II	No	**2.238**	**1.276**	**3.927**
Class III	No	0.543	0.245	1.203
Class IV	No	0.918	0.485	1.738

## Discussion

We observed a dramatically increased risk of IPD incidence in DIP compared to DM subjects. The HR was a little bit increased to 18.88 from 17.90 after adjustment by comorbidities and the causative drugs, which means the confounders did not contribute to the high risk of IPD incidence in DIP subjects.

The mean age of DIP subjects was 70.44 (9.43) and the incidence was increased by the age except for ≥ 80 years old group which showed an abrupt decrease. Savica et al reported the median age at onset of DIP was 70.9 years (interquartile range: 54.4–79.7) but they reported the incidence of DIP kept increased with older age [1]. The decreased DIP in the oldest group (≥ 80 years) might be attributable to the stringent inclusion criteria of this study. We excluded all subjects who had either DIP or IPD diagnosis codes in the previous 2-year period of enrollment to only include pure incidence DIP and IPD cases. So, the older adults who had those incidences already could not be included in our study ended up having a low proportion of DIP incidence in the oldest group. DIP incidence in females was higher than males in this study, which is in line with the results of the previous study [1].

In the DIP group, male to female IPD incidence ratio was 1.17 (15.58/13.24), which also agrees with the previous study. A review article which examined sex difference in IPD incidence reported IPD incidence ratio of male to female ranges from around 1.3 to 2.0 in most studies, but rates as low as 0.95 have been observed in Asia [28]. IPD incidence was the highest in the 60–69 year-old group, which is slightly different from previous Korean IPD epidemiology results in 2007 where a ceiling of IPD incidence in individuals aged 70–79 years was noted with a decreasing trend thereafter [29]. This factor implicates DIP diagnosis prompted more incident IPD in the younger group disrupting the natural aging course. In the DM group, IPD incidence was increasing with aging and reduced after 80 years old.

In Korea, females are known to have a higher incidence of both DIP [30] and IPD [31] than males, unlike what has been established in studies from other countries, where a higher male prevalence has been found for IPD and a higher female prevalence for DIP [1, 32]. However, in this study, females presented higher DIP but lower IPD incidence than males, implicating DIP incidence influenced more males resulting in higher IPD incidence in males than females.

In an attempt to evaluate the risk factors associated with IPD incidence in subgroup analysis, all comorbidities (dementia, depression, hypertension, and neurodegenerative disease) showed a trend for increased risks but with insignificant results.

The most well-known drugs associated with the development of DIP were typical antipsychotics but we did not observe a strong impact of antipsychotics on DIP incidence. This might be due to a trend to prescribe more atypical antipsychotics, which have a weaker D2 blocking effect than the first-generation ‘typical’ antipsychotics [9]. We found that in this study, Class I drugs which include antipsychotics showed the lowest incidence of IPD in the DIP group and it was not much high in the DM group as well. Classes II showed the highest incidence of IPD (27.69%), and III and IV showed a similar incidence (13.81 and 13.64%) in DIP subjects. CCB has been associated with the incidence of DIP out of plausible mechanisms that neurons increase their reliance on Ca (2+) channels to maintain autonomous activity with age. Blockage of Ca(2+) channels by CCB could pose sustained metabolic stress on mitochondria, accelerating dopaminergic neuronal cell death [33], In a series of DIP in older adults, about 2/3 of DIP incidences were caused by CCB and 70% presented tremor as the initial symptom [34]. Cinnarizine and flunarizine (only flunarizine was in the market in Korea during the study period), a selective T-type calcium channel blocker of which receptors are highly expressed in the basal ganglia, are used for vertigo and motion sickness. The risk can be twice as high for flunarizine compared with cinnarizine. The prevalence of parkinsonism with these drugs was found in 280 (3%) of 9,830 patients in a population-based study in Taiwan [35]. Whereas the most common classes of drugs reported in a pharmacovigilance study from France are antidepressants and antihistamines [32].

According to these findings, DIP incidence can be presumably a strong risk factor that alters the natural course of IPD development, resulting in features unlike those established in previous epidemiological analyses. However, we doubt that a causal relationship exists between DIP and IPD, as a few previous studies have proposed [14, 36]. If the toxic effects of the dopamine blocking-drugs caused irreversible damage to the neurons of vulnerable patients, then the potency and duration of the causative drugs taken by DIP subjects should have affected IPD incidence proportionately. However, we could not find support for this causal inference in our study. Instead, patients who had the lowest number of prescriptions of the causative drugs (duration: <30%) developed IPD the most often (17.89%). The strongest dopamine blockers, class I, were not associated with the highest number of IPD patients. From these observations, we conclude that DIP has been diagnosed in vulnerable patients who already had subclinical IPD pathology, and dopamine blockers acted as triggers unmasking IPD.

We suggest that our findings provide support for the theory that DIP diagnosis is a strong proxy indicator of risk for developing IPD. The causative drugs triggered IPD development from the existing subclinical Parkinsonism, as a few previous studies have suggested [7, 15].

Age-related decline or other environmental or traumatic insults have been resulted in damaging nigral dopaminergic neurons [37] and an increase in Lewy body fibrils (alpha-synuclein) [38]. These preconditions may remain in dormant or subclinical form and triggered by dopamine blockers [39]. Moreover, DIP diagnosis hastened the diagnosis (finding) of IPD by around 1-year, which has the strong clinical implication that clinicians should be alert to monitor the elderly who were diagnosed with DIP for potential IPD development around 1-year and be cautious in prescribing dopamine blockers to older adults.

We need to mention the advantage of selecting an active comparator to overcome healthy initiator bias commonly found in observational studies. This bias can arise through the selection of control groups with healthy and health-conscious patients, who through the effects of their healthy lifestyle, are also at a decreased risk of several adverse health outcomes [18, 19]. Therefore we selected DM patients as active comparators who might have a certain degree of increased risk of developing IPD [40].

We should address a few limitations of our study. Firstly, we only included 50-100- year-old population assuming the low IPD incidence in younger age might not affect the outcome. However, if we have included the younger population with DIP diagnosis, the patients might yield a moderate IPD outcome between two groups and give additional insights. Secondly, we classified antidopaminergic drugs based on their D2 receptor blocking potency. However, summing these agents into one single drug class might have some difficulties interpreting the outcomes considering their complex pharmacology. Thirdly, other risk factors of IPD such as dairy product consumption (urate-lowering effect) [41], smoking [42, 43]. and pesticide exposure [44] could not be assessed due to the data availability issue. We attempted to assess COPD (Chronic Obstructive Pulmonary Disease) as a proxy for smoking usually known for protective to IPD risk [45], but the number of subjects was too small to evaluate. Lastly, Parkinsonism may not have been recognized or diagnosed in our general population. For example, some mild symptoms related to DIP may not have been described in the medical records and had the diagnosis code because they were considered an inevitable consequence of the treatment with certain drugs (typical antipsychotics) resulting in an under-reporting of DIP [1].

## Conclusion

DIP was closely associated with the risk of IPD incidence. The association might not be causal rather DIP unmasks subclinical preexisting condition of IPD. Therefore, DIP diagnosis in older adults can be utilized as a strong proxy for IPD incidence.

## Supporting information

S1 Table Classification of DIP causing drugs as their potency for D2 receptor blockade. (DOCX)

S2 Table ICD-10 codes for comorbidities. (DOCX)

S3 Table Mean follow-up days and follow-up loss (death and DIP events). (DOCX)
