# Supplementary material for: Drug-induced Parkinsonism: A strong predictor of idiopathic Parkinson’s disease
Source: PLoS One. 2021 Mar 1;16(3):e0247354. doi: 10.1371/journal.pone.0247354 (PMC7920346; doi:10.1371/journal.pone.0247354)
Supplement: S1 Table — (DOCX) [file pone.0247354.s001.docx]

S1 Table. Classification of DIP causing drugs as their potency for D2 receptor blockade

| Class | Category | Drugs |
| --- | --- | --- |
| Class I | Dopamine D2 receptor blockers | Haloperidol, prochlorperazine, amisulpride, flupentixol, fluphenazine, levomepromazine, pimozide, promazine,  sulpiride, thioridazine, zuclopenthixol  risperidone, olanzapine, aripiprazole |
|  | Dopamine depleters | Tetrabenazine, reserpine, methyldopa |
|  | Dopamine synthesis blockers | Methyldopa |
| Class II | Calcium channel blockers (L-channel) | Verapamil, diltiazem |
|  | Calcium channel antagonists (p-channel) | Flunarizine |
| Class III | Antiemetics | Levosulpiride, itopride, clebopride, metoclopramide, prochlorperazine |
| Class IV | Antidepressants and others | Lithium, valproate, fluoxetine, sertraline, procainamide, amiodarone |
