# Supplementary material for: Drug-induced Parkinsonism: A strong predictor of idiopathic Parkinson’s disease
Source: PLoS One. 2021 Mar 1;16(3):e0247354. doi: 10.1371/journal.pone.0247354 (PMC7920346; doi:10.1371/journal.pone.0247354)
Supplement: S2 Table — (DOCX) [file pone.0247354.s002.docx]

**S2 Table. ICD-10 codes for comorbidities**

| **Comorbidities** | **ICD codes** |
| --- | --- |
| Dementia | F00.x, F01.x, F02.x, F03.x, F05.1, G30.x, G31.1 |
| Depression | F32.x, F33.x, F34.1, F41.2 |
| Neurodegenerative diseases | G213 G214 G22 G22.01 G230 G230.01 G231 G231.01 G232 G232.01 G233 G238 G238.01 G238.02 G239 G249.01 G251 G253.02 G254 G258 G258.02 G259 G26 G3100.02 G3182 G3183 G319 G111 G112 G12 F028.07 |
| Diabetes | E10, E11, E12, E13, E14 |

ICD-10: International Classification of Diseases, 10th Revision.
