# Supplementary material for: Drug-induced Parkinsonism: A strong predictor of idiopathic Parkinson’s disease
Source: PLoS One. 2021 Mar 1;16(3):e0247354. doi: 10.1371/journal.pone.0247354 (PMC7920346; doi:10.1371/journal.pone.0247354)
Supplement: S3 Table — (DOCX) [file pone.0247354.s003.docx]

| **Category** | | **DM (n=1,764)** | **DIP (n=441)** | ***P value**** |
| --- | --- | --- | --- | --- |
| Follow up period  (mean days **± SD**) | | 1208.66**±**485.78 | 1178.08**±**484.12 | 0.2369 |
| Follow-up loss  (Censoring) | Death | 446 | 77 | 0.00055 |
|  | DIP | 1 | - |  |
| IPD event | | 14 | 62 | <.00001 |

**S3 Table. Mean follow-up days and follow-up loss (death and DIP events)**

***Chi-square test**
